# Case Series of End-Stage Liver Disease Patients with Severe Coccidioidomycosis

**DOI:** 10.3390/jof9030305

**Published:** 2023-02-27

**Authors:** Daniel Ho, Kristen D. Kelley, Satya Dandekar, Stuart H. Cohen, George R. Thompson

**Affiliations:** 1 Department of Internal Medicine, University of California Davis, Sacramento, CA 95817, USA; 2 Department of Medical Microbiology and Immunology, University of California Davis, Davis, CA 95616, USA; 3 Department of Internal Medicine, Division of Infectious Diseases, University of California Davis, Sacramento, CA 95817, USA

**Keywords:** liver disease, hepatitis, *Coccidioides*, mortality

## Abstract

Liver disease causes relative compromise of the host immune system through multiple overlapping mechanisms and is an established risk factor for invasive fungal diseases including candidiasis and cryptococcosis. This immunologic derangement also leads to rapid progression of disease with resultant increases in morbidity and mortality. We describe severe coccidioidomycosis cases in the setting of liver dysfunction. Collaborative multi-center epidemiologic studies should be performed to determine the incidence of severe coccidioidomycosis in patients with concurrent liver disease.

## 1. Introduction

*Coccidioides* is a fungal pathogen endemic to the southwest United States, Mexico, and parts of Central and South America which may cause pulmonary or extrapulmonary infection known as coccidioidomycosis [1,2]. Infection is caused by the inhalation of spores from aerosolization when contaminated soil is disrupted, resulting in local disease in the lung or, in severe cases, dissemination to other major organs. Within the endemic regions, the risk of infection is approximately 3–5% per year; however, approximately 60% of cases are clinically asymptomatic or subclinical in nature [3]. Pulmonary involvement is the most common clinical presentation and in the endemic region it is estimated that approximately 15–29% of cases of community-acquired pneumonia are due to coccidioidomycosis [4,5]. Extrapulmonary manifestations or disseminated disease can involve the bone, skin, or central nervous system [6,7] and risk factors for disseminated disease often includes a variety of host and genomic factors [8,9]. Epidemiological studies show higher rates of disseminated disease in patients of African or Filipino ancestry [10], and rates of disseminated disease are also higher in patients with immunocompromising conditions such as hematologic malignancies, transplant patients on immunosuppressive therapy, and patients with human immunodeficiency virus (HIV).

End-stage liver disease (ESLD) causes relative compromise of the host immune system through multiple mechanisms and is an established risk factor for invasive candidiasis, cryptococcosis, and aspergillosis [11,12,13]. The association between ESLD and increased risk for other severe fungal infections, including coccidioidomycosis, has not previously been described. Herein, we report a case series of four patients seen at the UC-Davis Medical Center with ESLD identified by a review of patients with severe coccidioidomycosis at our center. These patients developed severe and rapidly progressive infection between 2017 and 2021, suggesting that ESLD could be associated with severe coccidioidomycosis. We call for additional epidemiologic studies in this field.

## 2. Cases

Case 1: A 49-year-old male with ESLD Child–Pugh class C from long standing co-infection with hepatitis B and hepatitis C presented to the emergency department with cough, chest pain and shortness of breath, and was found to have bilateral pneumonia. He was placed on broad-spectrum antibiotics, but over the ensuing 72 h developed respiratory failure requiring mechanical ventilation. He underwent bronchoscopy with subsequent cytology consistent with *Coccidioides* sp. (later confirmed by positive culture results), and he was started on liposomal amphotericin B (5 mg/kg/day). Over the following week, his blood cultures were positive for *Coccidioides* sp. And he further deteriorated and developed renal failure, hepatic encephalopathy, and disseminated intravascular coagulation (DIC). One month after the initial presentation, the patient succumbed to his illness due to refractory hypotension. Autopsy results confirmed severe *Coccidioides* infection throughout both lungs.

Case 2: A 56-year-old male with long standing hepatitis C infection and Child–Pugh class B cirrhosis presented to his primary care physician with acute complaints of a cough and oropharyngeal pain. His vital signs were normal except for an oxygen saturation of 90% in which he was subsequently admitted to a local hospital. Additional evaluation found a leukocytosis with significant eosinophilia and chest- X-ray consistent with bilateral pneumonia. He was started on empiric ceftriaxone, azithromycin, and fluconazole 800 mg daily with concern for acute pulmonary coccidioidomycosis. Four days after admission, he experienced respiratory decompensation despite supplemental oxygen, required mechanical ventilation and was diagnosed with acute respiratory distress syndrome (ARDS). Serologic studies for coccidioidomycosis returned positive for IgM by immunodiffusion and negative for IgG antibodies. Liposomal amphotericin (5 mg/kg/day) was added to his fluconazole; however, over the following week, he experienced persistent hypotension refractory to vasopressor support, developed renal failure and DIC. The patient died 3 weeks after initial presentation.

Case 3: A 35-year-old man with well-controlled HIV (CD4 count 855 cells/µL, HIV viral load undetectable on raltegravir and tenofovir disoproxil fumarate/emtricitabine presented to his primary care physician with new onset fever (38.4 °C), malaise, nausea, vomiting and unintentional weight loss of approximately ten pounds. He was admitted to a local hospital for additional evaluation. His laboratory values were significant for leukopenia and thrombocytopenia, and the initial X-ray was unrevealing; however, computed tomographic imaging showed diffuse interstitial densities throughout both lungs and evidence of hepatic cirrhosis and moderate ascites. He was found to have hepatitis C (Child–Pugh class B). The patient underwent a diagnostic bronchoscopy and was treated for community acquired pneumonia with levofloxacin 500 mg daily. His fever persisted, and on day fourteen, his admission blood cultures returned positive for mold consistent with *Coccidioides* sp. The patient was started on fluconazole 800 mg daily; however, over the next four days, he continued to worsen and required mechanical ventilation and died later the same day due to refractory hypotension. Autopsy results found disseminated coccidioidomycosis with histopathologic findings consistent with *Coccidioides* sp. In the lung, ascitic fluid, and throughout the mediastinal and hilar lymphatic tissue.

Case 4: A 43-year-old man with untreated hepatitis C and Child–Pugh class C cirrhosis complicated by hepatic encephalopathy and portal hypertension presented to his primary care physician to discuss hepatitis C treatment and was noted to have fever upon intake (38.1 °C). He underwent examination and was found to have scant rales, and a subsequent chest X-ray found a right lower lobe consolidation. He was admitted to a local hospital and additional radiographic imaging found bilateral interstitial pneumonia and disseminated coccidioidomycosis with lesions in multiple ribs and throughout the lumbar spine, confirmed as coccidioidomycosis by histopathology and culture. He was treated with liposomal amphotericin B (5 mg/kg/day) and fluconazole (600 mg) daily. He continued to deteriorate with persistent fever, worsening pneumonia, and developed new onset renal failure requiring hemodialysis. He subsequently developed hypotension and required mechanical ventilation due to lethargy. Despite supportive measures, his hypotension worsened and on day 31 the patient died from his illness.

## 3. Discussion

The majority of cases of coccidioidomycosis are asymptomatic or, if clinically apparent, typically contained within the pulmonary system. Severe disseminated disease can occur particularly in immunocompromised patients, including those with hematologic malignancy, bone marrow or solid organ transplant recipients, or patients with HIV. ESLD is increasingly recognized as an immunocompromising condition and is a well-recognized and significant risk factor for severe fungal disease [11,12].

The relative immune system dysfunction in which patients with ESLD may be predisposed to severe fungal infections is referred to as cirrhosis-associated immune dysfunction. Liver impairment results in abnormalities of both the innate and adaptive systems, resulting in impaired immune surveillance from reduced synthesis of molecules essential for mounting an adequate immune response [14]. One of these mechanisms in which liver dysfunction disrupts immune surveillance is via decreased efficacy of the liver resident macrophages (Kupffer cells), which make up approximately 90% of the body’s macrophages as a part of the reticuloendothelial system. Kupffer cells are known to play a vital role in the clearance of bacteria and endotoxins from the bloodstream. Prior studies have illustrated the efficiency of this system with radiolabeled intravenously administered Gram-negative rods cleared by the liver within 10 min [15]. Similarly, Kupffer cells have been found to capture circulating *Cryptococcus* and *Candida* spp preventing dissemination to other organs [16]. In ESLD portosystemic shunting of blood due to underlying portal hypertension and abnormal liver architecture causes a net reduction in blood flow to the liver, further impairing Kupffer cell clearance of circulating pathogens. This leads to increased systemic exposure to circulating fungal organisms increasing the risks for severe and/or disseminated infection.

Immunologic impairment in liver disease also results from the reduction in protein synthesis required for a functioning immune system with resultant relative complement deficiencies, impaired ability for opsonization, and decreased neutrophil phagocytosis–all essential facets of *Coccidioides* recognition [12,17,18]. The synthesis of numerous acute phase reactants also assisting in opsonization is also impaired in cases of liver dysfunction. In ESLD, neutrophils are less activated and are functionally deficient with decreases in chemotaxis, adherence, and phagocytosis, along with other circulating immune cells including monocytes, B lymphocytes, T lymphocytes, and natural killer lymphocytes [14]. Additionally the hypersplenism often present in liver disease can produce quantitative reductions in circulating white blood cells, including CD4+ T cells required for the control of most fungal pathogens [19].

These immunologic derangements in liver disease are predisposing factors for *Candida*, *Cryptococcus* and *Aspergillus* spp and clearly elevate patient risk for these infections. For example, although esophageal candidiasis is traditionally seen in HIV patients, or non-HIV patients with malignancy, diabetes, gastric surgery, or medications, a retrospective review demonstrated ESLD should be considered a risk factor for esophageal candidiasis given the incidence in patients with cirrhosis was significantly higher compared to non-cirrhotic patients [20].

The lack of an appropriate immune response also reduces patient symptoms, and they may thus present with later stages of the disease increasing subsequent morbidity and mortality. Those with ESLD who develop invasive candidiasis have higher odds of death, intensive care unit admission, and mortality (up to 54.7% increasing linearly with MELD score) [21]. Cryptococcal infection in patients with liver disease also has higher associated mortality compared with other *Cryptococcus*-infected populations [11].

Treatment in patients with ESLD secondary to invasive fungal infections is often difficult as the triazoles and lipid amphotericin B formulations may all cause hepatotoxicity and exhibit variable pharmacokinetics in this patient population [22]. Although these classes are recommended for severe coccidioidomycosis, caution and close monitoring is warranted in those with hepatic impairment.

The presentation and rapid decline in patients with underlying liver disease and coccidioidomycosis presented here is unusual as multiorgan failure, shock, and death are uncommon outcomes in coccidioidal infection. The relative immunodeficiency seen in liver disease likely played a key role in the precipitous decline described in this report. ESLD is considered an immunocompromising condition due to a variety of mechanisms of liver dysfunction resulting in an abnormal immune system response in both the innate and adaptive systems. We propose that coccidioidomycosis be added to the list of fungal infections with rapid and/or severe outcomes in patients with underlying liver disease. Larger collaborative multi-center epidemiologic studies should be performed to confirm this observation.
